# Supplementary material for: Predictive value of physical and blood examination findings for short-term mortality in dogs with respiratory disorders
Source: PLoS One. 2025 Jul 17;20(7):e0328797. doi: 10.1371/journal.pone.0328797 (PMC12270126; doi:10.1371/journal.pone.0328797)
Supplement: S1 Table — The table shows variables that were included at the beginning of the multivariate logistic regression model but were excluded during backward stepwise selection based on the Akaike information criterion. These values such as odds ratio and P value should be interpreted with caution, as they are model-dependent and do not reflect the final model output. (DOCX) [file pone.0328797.s001.docx]

| Variable | Odds ratio | 95% CI | P value |
| --- | --- | --- | --- |
| HR (per min) | 1.04 | 0.93 – 1.16 | 0.525 |
| BT (℃) | 1.58 | 0.32 – 7.93 | 0.578 |
| WBC (×10^3^/μL) | 1.00 | 1.00 – 1.00 | 0.513 |
| Glu (mg/dL) | 0.99 | 0.95 – 1.02 | 0.538 |
| Phosphate (mg/dL) | 4.36 | 0.79 – 2.40 | 0.090 |
| Lactate (mmol/L) | 1.47 | 0.82 – 2.64 | 0.192 |
