# Supplementary material for: Predictive value of physical and blood examination findings for short-term mortality in dogs with respiratory disorders
Source: PLoS One. 2025 Jul 17;20(7):e0328797. doi: 10.1371/journal.pone.0328797 (PMC12270126; doi:10.1371/journal.pone.0328797)
Supplement: S2 Table — (DOCX) [file pone.0328797.s002.docx]

| Variable | n | Survivors | n | Non-survivors | P value |
| --- | --- | --- | --- | --- | --- |
| Age (years) | 37 | 12.0 (5.0 – 15.7) | 11 | 12.1 (9.1 – 14.0) | 0.184 |
| Sex (n) | 37 | male：16 female：21 | 11 | male：4 female：7 | 0.741 |
| BW (kg) | 37 | 3.30 (1.58 - 10.65) | 11 | 3.20 (1.88 - 4.7) | 0.990 |
| BCS (1 to 5) | 20 | 2 (2 - 3) | 5 | 2 (2 - 4) | 1 |
| Temperature (℃) | 32 | 37.9 (35.2 - 39.2) | 8 | 37.0 (34.9 - 38.0) | 0.006 |
| Heart rate (per min) | 29 | 156 (84 - 210) | 6 | 130 (84 - 156) | 0.058 |
| Respiratory rate (per min) | 22 | 98 (32 - 156) | 8 | 62 (36 - 150) | 0.312 |
| Cardiac murmur (1 to 6) | 28 | 4 (3 - 6) | 8 | 4 (3 - 5) | 0.408 |
| WBC (×10^3^/μL) | 25 | 14730 (7280 - 35620) | 10 | 19225 (11260 - 39030) | 0.162 |
| Platelets ((×10^3^/μL) | 25 | 411 (238 - 770) | 10 | 372 (122 - 640) | 0.358 |
| PCV (%) | 25 | 43.9 (32.1 - 51.3) | 10 | 42.4 (35.3 - 60.0) | 0.812 |
| Glu (mg/dL) | 19 | 108 (93 - 243) | 10 | 152 (59 - 419) | 0.041 |
| Albumin (g/dL) | 23 | 2.9 (2.2 - 3.5) | 9 | 3 .1(2.6 – 4.0) | 0.184 |
| BUN (mg/dL) | 24 | 29 (13 - 67) | 10 | 29 (12 - 130) | 0.650 |
| Creatinine (mg/dL) | 24 | 0.9 (0.3 - 1.8) | 10 | 0.7 (0.4 - 5.5) | 0.303 |
| Calcium (mg/dL) | 16 | 9.3 (7.9 - 10.8) | 8 | 9.0 (7.3 - 9.9) | 0.341 |
| Phosphate (mg/dL) | 16 | 5.1 (3.0 - 8.5) | 8 | 6.8 (4.6 - 12.3) | 0.006 |
| ALT (U/L) | 20 | 86 (19 - 282) | 8 | 64 (40 - 113) | 0.321 |
| ALP (U/L) | 20 | 89 (10 - 981) | 9 | 83 (25 - 542) | 1 |
| Total bilirubin (mg/dL) | 13 | 0.3 (0.1 - 0.4) | 9 | 0.1 (0.1 - 3.4) | 0.630 |
| Total cholesterol (mg/dL) | 13 | 149 (79 - 247) | 9 | 156 (56 - 325) | 0.124 |
| Sodium(mEq/L) | 25 | 153 (147 - 162) | 9 | 149 (124 - 158) | 0.164 |
| Potassium(mEq/L) | 25 | 4.3 (3.1 - 5.4) | 10 | 5.1 (4.0 - 5.3) | 0.082 |
| Chloride(mEq/L) | 25 | 115 (105 - 125) | 10 | 113 (88 - 118) | 0.078 |
| CRP(mg/dL) | 26 | 1.75 (0.05 - 20) | 10 | 0.45 (0 - 5.5) | 0.458 |
| Lactate (mmol/L) | 10 | 1.9 (4.8 – 8.0) | 4 | 4.8 (1.9 – 8.0) | 0.177 |
