# Supplementary material for: Predictive value of physical and blood examination findings for short-term mortality in dogs with respiratory disorders
Source: PLoS One. 2025 Jul 17;20(7):e0328797. doi: 10.1371/journal.pone.0328797 (PMC12270126; doi:10.1371/journal.pone.0328797)
Supplement: S3 Table — (DOCX) [file pone.0328797.s003.docx]

| Variable | n | Survivors | n | Non-survivors | P value |
| --- | --- | --- | --- | --- | --- |
| Age (years) | 68 | 10 (0.2 - 17) | 17 | 12.0 (0.1 – 15.5) | 0.956 |
| Sex (n) | 68 | male：38 female：30 | 17 | male：6 female：11 | 0.176 |
| BW (kg) | 68 | 4.38 (0.64 - 29.15) | 17 | 4.56 (0.433 - 16.6) | 0.648 |
| BCS (1 to 5) | 33 | 3 (1 - 5) | 11 | 3 (2 - 5) | 0.499 |
| Temperature (℃) | 66 | 38.7 (35.9 - 40.7) | 15 | 37.4 (34.1 - 40.3) | <0.001 |
| Heart rate (per min) | 40 | 135 (72 - 240) | 13 | 120 (60 - 192) | 0.209 |
| Respiratory rate (per min) | 33 | 56 (20 - 126) | 10 | 94 (30 - 156) | 0.077 |
| Cardiac murmur (1 to 6) | 66 | 0 (0 - 5) | 15 | 0 (0 - 3) | 0.010 |
| WBC (×10^3^/μL) | 49 | 12820 (3690 - 48390) | 16 | 20855 (6860 - 51740) | 0.007 |
| Platelets ((×10^3^/μL) | 49 | 354 (71 - 1173) | 16 | 438 (145 - 801) | 0.088 |
| PCV (%) | 48 | 43.0 (26.1 - 53.9) | 16 | 40.0 (23.7 - 70.5) | 0.871 |
| Glucose (mg/dL) | 36 | 103 (75 - 158) | 16 | 133 (91 - 446) | 0.004 |
| Albumin (g/dL) | 35 | 3.1 (2.5 - 4.3) | 14 | 2.9 (2.1 – 3.7) | 0.012 |
| BUN (mg/dL) | 38 | 20 (7 - 52) | 15 | 25 (12 - 72) | 0.018 |
| Creatinine (mg/dL) | 38 | 0.8 (0.3 - 1.8) | 15 | 0.8 (0.3 - 1.4) | 0.976 |
| Calcium (mg/dL) | 27 | 9.7 (7.9 - 12.8) | 13 | 9.0 (8.1 - 12.0) | 0.169 |
| Phosphate (mg/dL) | 28 | 4.4 (2.8 - 7.8) | 13 | 6.5 (4.6 - 9.4) | <0.001 |
| ALT (U/L) | 36 | 61 (18 - 762) | 14 | 67 (26 - 182) | 0.627 |
| ALP (U/L) | 35 | 130 (25 - 490) | 14 | 126 (49 - 528) | 0.877 |
| Total bilirubin (mg/dL) | 26 | 0.2 (0.1 - 1.6) | 12 | 0.3 (0.1 - 0.5) | 0.774 |
| Total cholesterol (mg/dL) | 26 | 174 (114 - 242) | 13 | 197 (127 - 411) | 0.098 |
| Sodium(mEq/L) | 37 | 154 (147 - 163) | 15 | 155 (143 - 166) | 0.395 |
| Potassium(mEq/L) | 39 | 4.6 (3.7 - 5.5) | 15 | 4.1 (3.3 - 5.1) | 0.061 |
| Chloride(mEq/L) | 39 | 116 (109 - 121) | 15 | 115 (102 - 123) | 0.831 |
| CRP (mg/dL) | 49 | 2 (0-20) | 13 | 8.6 (0.1-20) | 0.202 |
| Lactate (mmol/L) | 4 | 2.4 (1.7 – 13.3) | 9 | 8.0 (2.4 – 19.8) | 0.142 |
