# Supplementary material for: Predictive value of physical and blood examination findings for short-term mortality in dogs with respiratory disorders
Source: PLoS One. 2025 Jul 17;20(7):e0328797. doi: 10.1371/journal.pone.0328797 (PMC12270126; doi:10.1371/journal.pone.0328797)
Supplement: S4 Table — (DOCX) [file pone.0328797.s004.docx]

| Variable | Odds ratio | 95% CI | P value |
| --- | --- | --- | --- |
| Albumin (g/dL) | 0.027 | 0.0005 – 1.38 | 0.072 |
| Phosphate (mg/dL) | 5.98 | 1.87 – 19.20 | 0.003 |
