# Supplementary material for: Predictive value of physical and blood examination findings for short-term mortality in dogs with respiratory disorders
Source: PLoS One. 2025 Jul 17;20(7):e0328797. doi: 10.1371/journal.pone.0328797 (PMC12270126; doi:10.1371/journal.pone.0328797)
Supplement: Attachment — Submitted filename: Response to Reviewers.docx [file pone.0328797.s006.docx]

Dr. Tomasz W. Kaminski

Academic Editor

PLOS ONE

May 14, 2025

Dear Dr. Kaminski,

Re: Resubmission of manuscript reference No. PONE-D-25-03766

Thank you for the opportunity to revise and resubmit our manuscript entitled

"Findings of physical examination and blood test are related to poor prognosis in canine respiratory disorders: a retrospective evaluation of 113 dogs (2016-2019)."

We appreciate the thoughtful and constructive feedback provided by the reviewers and editorial team. We have carefully addressed all comments and have substantially revised the manuscript accordingly.

Firstly, in accordance with the first comment of Reviewer 2, the title has been revised as "Predictive value of physical and blood examination findings for short-term mortality in dogs with respiratory disorders."

Secondly, we identified some inconsistencies in terminology and expressions within the main text and have made minor revisions to improve consistency, without altering the overall structure or conclusions of the study. In addition, typographical errors were found in Table 1,3,4, and 5. These errors have been corrected and are limited to the tables themselves, with no impact on the study results or subsequent statistical interpretations.

Below, we present each reviewer's comments and our response to it.:

Reviewer #1:

Comment: **To help this study add additional context to these findings, I would like for the authors to expand on their discussion of the increased in phosphate; and more specifically, the therapeutic interventions that generally are used for hyperphosphatemia and how that may impact outcome. Or the authors could expand on the additional testing factors (i.e. hemoconcentrations) which could dive into a deeper understanding of the clinical mechanisms observed.**

Response: Thank you for this insightful comment. We have expanded the Discussion section to include detailed descriptions of potential mechanisms by which elevated phosphate levels may contribute to tissue injury, including mineralization of soft tissues and oxidative DNA damage in lung epithelial cells. Furthermore, therapeutic interventions are also mentioned in the same paragraph.

Revised text (Lines 309–320): “While this study focused on plasma phosphate concentration as a prognostic indicator, elevated phosphate levels are also known to act as a pathophysiological factor that contributes directly to tissue injury. One well-established mechanism involves ectopic mineralization of soft tissues, including the cardiovascular system and kidneys [32]. Additionally, recent evidence suggests that hyperphosphatemia may directly damage pulmonary tissue by inducing oxidative DNA damage and promoting apoptosis in lung epithelial cells [33]. Although various therapeutic interventions for hyperphosphatemia, such as phosphate binders or dietary phosphorus restriction, are used in clinical practice, their efficacy in the context of acute respiratory disorders in dogs remains unclear. Therefore, further studies are needed to clarify not only the role of phosphate elevation in pulmonary injury, but also whether therapeutic management of hyperphosphatemia may improve outcomes in canine respiratory disorders.”

Reviewer #2:

Comment: **The title is overly long and could be more concise.**

Response: We appreciate the reviewer’s suggestions. As suggested, we have changed the title as " Predictive value of physical and blood examination findings for short-term mortality in dogs with respiratory disorders."

Comment: **Line 24 “Introduction” This line header is fine and serves its purpose, though unnecessary in an abstract**

**Lines 25–29 The objective is vague and lacks specificity. The phrase "utility of screening test as severity assessment" is unclear. Does the study aim to validate a prognostic score, or simply find statistically significant associations?**

Response: We appreciate these helpful suggestions. We have revised the abstract to better communicate our objectives to readers.

Revised text (Lines 25–28): “ Background. Similar to human medicine, attempts have been made in veterinary medicine to assess the severity of respiratory disorders using methods other than respiratory function evaluation; however, such approaches remain insufficient.”

Comment: **Line 52 Opens with a generic statement lacking scientific focus. It would be stronger to immediately frame the importance of early prognostic indicators in acute respiratory presentations.**

Response: We thank the reviewer for this constructive suggestion. In the revised Introduction, we have restructured the opening to more clearly highlight the clinical importance of early prognostic evaluation in dogs presenting with acute respiratory distress. We now begin by emphasizing the need for rapid and reliable risk stratification in veterinary emergency care.

Revised text (Lines 51–55): Respiratory disorders are among the most common causes of emergency presentations in small animal practice [1]. These conditions often progress rapidly, leading to severe hypoxemia, and therefore require prompt and accurate initial assessment. Prolonged hypoxia may result in multi-organ dysfunction syndrome, highlighting the importance of systemic evaluation in addition to respiratory function assessment [2].

Comment: **Lines 60-67 The reference to the PSI is a reasonable intuition, but the analogy from human medicine to canine patients is merely implied rather than substantiated. It is not clarified whether such a scoring tool exists in veterinary medicine, or whether this study aims to propose one.**

**Moreover, the statement that general clinical parameters are more predictive than measures of oxygenation is made without discussing the scope or validity of this claim (PSI is designed specifically for community-acquired pneumonia).**

**The authors should more clearly define the existing gap in veterinary medicine. Are similar prognostic tools already available? Have there been previous attempts to develop one? Is the purpose of this study to address a specific unmet need, or simply to explore associations between selected parameters?**

**Lines 79–81 The assertion that the usefulness of screening tests is unknown is overstated. There are multiple veterinary studies evaluating CBC/chemistry values as prognostic tools in critical care.**

Response: We appreciate this important point. We have revised the paragraph to explicitly state that while scoring tools like the Pneumonia Severity Index (PSI) are well-established in human medicine, there are currently no widely accepted equivalent scoring systems in veterinary respiratory medicine. We now clarify that our study does not aim to develop a scoring tool but rather to explore whether commonly obtained physical and blood examination findings are associated with short-term outcomes in dogs with radiographically confirmed respiratory disorders. We have also moderated the statement regarding general parameters versus oxygenation measures and clarified that this conclusion is based on prior PSI studies in humans, not intended to be directly generalized to veterinary patients.

Moreover, considering that previous studies have evaluated CBC and serum chemistry parameters in veterinary critical care settings, we have revised this sentence to reflect that while certain studies have investigated individual parameters, the prognostic value of a combination of physical and routine blood examination findings specifically in dogs with respiratory disorders remains unclear. This clarification has been added to better reflect the existing literature.

Revised text (Lines 68–77): “In contrast, veterinary medicine currently lacks disease-specific scoring systems for respiratory disorders. Prognostic evaluation is primarily based on respiratory function parameters such as SpO₂ and arterial blood gas analysis. General health assessment in dogs typically involves physical examination, blood tests, urinalysis, ultrasonography, and radiography; however, few studies have investigated the prognostic value of these data in canine respiratory disorders. For instance, a previous study reported that age, mental status, hypoalbuminemia, and elevated BUN were associated with outcomes in dogs with aspiration pneumonia [5]. Meanwhile, another study showed that total white blood cell count and neutrophil-to-lymphocyte ratio were not useful prognostic markers in canine pneumonia [6].”

Comment: **Lines 89-91 define a hypothesis: i.e., "We hypothesized that abnormalities in commonly available physical and blood parameters would be associated with increased short-term mortality in dogs with radiographically confirmed respiratory disease."**

Response: We appreciate this suggestion. As recommended, we have now explicitly stated our hypothesis at the end of the Introduction.

Revised text (Lines 68–77): “While efforts have been made to identify prognostic indicators beyond respiratory parameters, sufficient validation is lacking. Therefore, in this study, we hypothesized that abnormalities in commonly available physical and blood parameters would be associated with increased short-term mortality in dogs with radiographically confirmed respiratory disorders, and we aimed to investigate the feasibility of this approach.”

Comment: **Lines 96–100 Selection criteria are vague. How was the diagnosis confirmed? Was there a standard diagnostic approach (e.g., radiographic criteria)? Was the diagnosis made by board-certified radiologists?**

**Lines 100–102 The use of a questionnaire sent to referring veterinarians post-discharge introduces recall and reporting bias. There’s no explanation of the response rate or how non-responders were handled.**

**Line 102-104 The exclusion criteria raise several concerns that should be addressed for clarity and methodological rigor. “Cases without radiographic abnormalities in respiratory organs” This suggests that only patients with visible radiographic changes were included. However, this may systematically exclude certain respiratory conditions (e.g., early-stage tracheal collapse, laryngeal paralysis, or functional respiratory disorders) where radiographs may appear normal. The authors should justify this decision and discuss the potential for selection bias.**

**“Cases having no relationship between symptoms and radiographic findings” This is vague and subjective. How was the “relationship” assessed? Who determined whether clinical signs matched radiographic findings? The criterion is unclear.**

Response: In the revised Materials and Methods section, we have clarified that radiographic evaluations were performed by veterinarians with sufficient clinical experience. To ensure consistency in case selection, we included only cases with radiographic abnormalities and for which follow-up data were available. As the reviewer correctly pointed out, this criterion may have resulted in the exclusion of functional respiratory disorders or early-stage lesions with no visible radiographic changes. We have now addressed this potential for selection bias in the Limitations section. Additionally, we have now stated the number of cases excluded due to unavailable follow-up beyond 7 days in the Results section. Furthermore, we acknowledge that the use of questionnaires introduces potential sources of bias, such as recall and reporting bias, and we have now explicitly mentioned these in the Limitations section.

Revised text (Lines 96–98): “All radiographic findings were evaluated by the attending emergency veterinarian with at least 10 years of clinical experience, and the radiographic features were reviewed by first author.”

Revised text (Lines 358–362): “Firstly, only cases with radiographically confirmed respiratory abnormalities were included. As a result, certain conditions such as functional disorders or early-stage diseases without apparent radiographic changes may have been excluded. However, this criterion was considered necessary measures to maintain diagnostic consistency and to enhance the reliability of comparative analysis.”

Revised text (Lines 152–153): “The response rate to the follow-up questionnaire was 60.9 % (137/225 cases).”

Revised text (Lines 362–368): “Second, the use of follow-up surveys to obtain clinical outcomes may introduce bias. The response rate was approximately 60%, which may have led to selection bias, as cases with incomplete follow-up data were excluded from the analysis. Since outcome data were not directly collected at the study institution, misclassification or underreporting of adverse outcomes cannot be completely ruled out. Future prospective studies with standardized follow-up protocols are warranted to minimize these biases.”

**“Cases without follow-up data after 7 days” Excluding all cases without 7-day follow-up could introduce significant selection bias, especially if lost cases differ systematically from those retained (e.g., healthier animals discharged early or euthanized without veterinary follow-up). The authors should report how many cases were excluded for this reason and whether their baseline characteristics differed from included cases. Moreover, the grouping of dogs into “Survivors” and “Non-survivors” based on a 7-day window is arbitrary and not justified with clinical rationale or references. Please explain the rationale for excluding dogs without follow-up. This could introduce selection bias if dogs that recovered quickly or were lost to follow-up had different clinical characteristics.**

**Line 163–165 96.4% of non-survivors died within 24 hours. This suggests the "Non-survivor" group is dominated by peracute presentations, making comparisons with survivors (who lived >7 days) potentially invalid.**

Response: Regarding the 7-day follow-up period, we have clarified in the manuscript that our objective was to evaluate short-term prognosis in canine respiratory disorders. Following precedents from previous studies on acute and severe illnesses, we defined short-term outcome as survival within 7 days. Considering that the majority of non-survivors in our study died within 24 hours, we believe this classification is appropriate for capturing acute-phase outcomes.

Revised text (Lines 107–110):” In this study, based on previous study, short-term prognosis was defined as 7 days, and dogs that survived more than 7 days from admission were classified as the Survivors, while those that died naturally or were euthanized within 7 days were classified as Non-survivors [8].”

Revised text (Lines 153–155):” Four cases were excluded due to the lack of follow-up information from the referring veterinarians. Consequently, the remaining 133 dogs (59.1%) were included in the final analysis (Fig.1).”

Comment: **Lines 118-119 The only physical examination findings considered were body weight, heart rate HR, respiratory rate, body temperature and heart murmur but detail on respiratory and cardiovascular clinical parameters at presentation are not provided (mucous membrane color, jugular vein distension or pulsation, pulse quality, respiratory pattern, effort of breathing, respiratory sounds, presence/absence of cough, orthopnea). In my opinion, for a study aimed at identifying prognostic indicators in canine respiratory disorders, the absence of these variables is a significant limitation.**

**Furthermore, the absence of any discussion regarding cardiac disease is a significant omission. Cardiogenic pulmonary edema is one of the most common causes of respiratory distress in dogs, and differentiating it from primary respiratory conditions is a critical aspect of case management. I am sure that cardiovascular disease was accurately ruled out. However, without this information, the reader cannot assess the case mix, nor understand the relevance of the prognostic indicators identified.**

Response: We appreciate this important observation. As this was a retrospective study, not all physical examination variables (e.g., mucous membrane color, pulse quality, respiratory effort) were consistently recorded and thus were excluded from analysis. We have added this clarification to the revised manuscript. Regarding cardiac disease, we have now performed a subgroup analysis separating cases with cardiogenic pulmonary edema (CPE) from non-CPE, using clinical signs, radiographic features, echocardiographic findings (when available), and history to define the CPE group. The results of this post hoc analysis showed that phosphate remained a significant predictor in both groups (Results section, Table S2–S4). These additions have been integrated and discussed appropriately.

Revised text (Lines 368–371):” Third, although additional clinical variables such as respiratory pattern or effort of breathing were often assessed during examination, these parameters were inconsistently recorded and therefore excluded from statistical analysis. Prospective studies are warranted to evaluate their prognostic value.”

Revised text (Lines 220–239):” Since the results might have been influenced by the presence or absence of cardiac disease, a post hoc subgroup analysis was performed by dividing the cases into two groups: those with cardiogenic pulmonary edema (CPE), which is a common cause of respiratory distress in dogs, and those with other respiratory disorders (non-CPE). 48 cases were clinically suspected to have cardiogenic origin based on concurrent findings of heart murmur, radiographic features, echocardiographic parameters, or history of cardiac disease. [9-11] Univariate analysis showed that in dogs with CPE, BT was significantly lower, and Glu and phosphate concentration were significantly higher in Non-survivors than in Survivors (S2 table). Multivariable logistic regression was attempted using these variables; however, due to a high rate of missing data in the Non-survivors, only 5 cases were available for analysis. In addition, strong collinearity was observed among the variables (VIF > 10). Although we applied stepwise selection using the Akaike information criterion to reduce the number of predictors, the model failed to converge, and multivariable analysis could not be performed. Therefore, only the results of univariable analyses are reported for the CPE group. In contrast, among dogs in the non-CPE group, univariate analysis revealed that BT and Albumin were significantly lower and WBC, Glu, BUN and blood phosphate were significantly higher in Non-survivors compared to Survivors (S3 table). Multivariable logistic regression showed only phosphate remained a statistically significant variable in the logistic regression analysis in non-CPE group (S4 table). These findings were consistent with the results from the overall and lung parenchymal disorder groups”

Revised text (Lines 255–259):” Additionally, our subgroup analysis of dogs with CPE and other diseases revealed similar trends in clinical parameters as seen in the overall population. Notably, blood phosphate concentration remained a significant prognostic indicator in multivariate analysis. These findings reinforce the utility of phosphate as a general prognostic biomarker across diverse etiologies of respiratory disorders.”

Comment: **Line 129–139 The description of the statistical analysis is superficial. No mention of power calculation, model diagnostics, or handling of missing data.**

Response: We thank the reviewer for this valuable comment. We have revised the Methods section to provide more detail on our statistical approach. Cases with missing values in any of the variables used for multivariate analysis were excluded using listwise deletion. In this study, given the retrospective design, no a priori power calculation was performed. However, to reduce overfitting, we used backward stepwise logistic regression based on AIC.

Revised text (Lines 127–132):” To avoid model overfitting due to the limited sample size, the models were designed via backward elimination and optimized using the Akaike information criterion. Cases with missing values in any of the variables used for multivariate analysis were excluded using listwise deletion, in accordance with the default setting of the statistical software. Therefore, only complete cases were included in the logistic regression analysis.”

Comment: **Lines 174–176 Multivariate analysis yields phosphate as the only significant variable. In Table 4 please include OR and 95% CIs even if not retained in the final model.**

Response: As suggested, we have added a supplementary table (Table S1) that includes odds ratios and 95% confidence intervals for all variables entered into the multivariate model, including those excluded during stepwise selection. We clarified this addition in the Results section and referenced it appropriately.

Revised text (Lines 176–177):” Other variables that were initially included in the model but excluded during the stepwise selection process are presented in S1 table.”

Comment: **Lines 240–261 The mechanism by which phosphate increases mortality is unclear. The authors speculate about AKI and tissue hypoxia, but provide no evidence of renal failure or acid-base disturbances in these dogs.**

Response: We have revised the Discussion section to clarify that while we speculate on possible mechanisms such as AKI or hypoxia-induced phosphate shifts, our study lacks direct measures of renal function or acid-base status. We now explicitly state this limitation and cite literature suggesting potential mechanistic pathways (Lines 299–311). We emphasize that the proposed mechanisms remain hypothetical and that further mechanistic studies are warranted.

Revised text (Lines 306–308):” Unfortunately, while elevated phosphate levels were statistically associated with poor outcome, the underlying mechanisms remain speculative due to the lack of confirmatory data. This warrants further mechanistic studies.”

Comment: **Lines 288–295 The discussion of BUN and WBC as correlates of poor outcome is consistent with critical care literature. However, the decision to exclude these from the final model should be discussed.**

Response: We agree and have addressed this in the Discussion. While BUN and WBC were significantly different in univariate analysis, they were excluded during stepwise model selection, likely due to multicollinearity or weaker independent predictive value. Therefore, we added a brief interpretation of why phosphate may have remained while others did not.

Revised text (Lines 350–357):” To avoid model overfitting due to the limited sample size, we used a backward stepwise logistic regression model based on the Akaike Information Criterion to reduce the number of explanatory variables. Although variables such as BUN, WBC and Glu were significantly different in the univariate analysis, they were not retained in the multivariate model. This may reflect inter-variable correlations and the limited sample size, which reduced the statistical power for detecting independent associations. Therefore, in order to create a more accurate model, it is necessary to increase the number of samples and conduct further studies.”

Comment: Lines **311–317 The potential confounding effect of euthanasia is acknowledged only minimally. There is no analysis separating dogs who died naturally from those euthanized.**

Response: Thank you for this comment. We have clarified in the revised manuscript that only one dog in the non-survivor group was euthanized and that its phosphate level exceeded the cut-off value, suggesting it did not bias the model's predictive power. This is now explicitly stated in the Limitations section.

Revised text (Lines 218–219):” This study included one euthanasia case, and even in this case the phosphorus level was higher than the cut-off value.”

Revised text (Lines 372–377):” Fourth, the inclusion of euthanized dogs in Non-survivors may have introduced a confounding effect, as euthanasia decisions may have been influenced by factors other than clinical severity, such as owner preference or financial considerations. However, only one dog was euthanized in this study, and its plasma phosphate concentration exceeded the identified cut-off value, suggesting that the impact of euthanasia on the overall results was likely minimal.”

We believe these revisions have significantly improved the clarity and scientific rigor of the manuscript. We respectfully request your favorable reconsideration of our work for publication in PLOS ONE. Please feel free to contact us should you require any further information.

Sincerely,

Toshiyuki Tanaka, DVM, PhD

Osaka Metropolitan University

Laboratory of Veterinary Advanced Diagnosis and Treatment

1-58, Rinku-oraikita, Osaka city, Osaka, 598-8531, Japan

email: f21724w@omu.ac.jp
