# Supplementary material for: Predictive value of physical and blood examination findings for short-term mortality in dogs with respiratory disorders
Source: PLoS One. 2025 Jul 17;20(7):e0328797. doi: 10.1371/journal.pone.0328797 (PMC12270126; doi:10.1371/journal.pone.0328797)
Supplement: Attachment — Submitted filename: Response to Reviewers-2.docx [file pone.0328797.s007.docx]

Dr. Tomasz W. Kaminski

Academic Editor

PLOS ONE

July 5, 2025

Dear Dr. Kaminski,

Re: Resubmission of manuscript reference No. PONE-D-25-03766

Thank you for the opportunity to revise and resubmit our manuscript entitled

" Predictive value of physical and blood examination findings for short-term mortality in dogs with respiratory disorders."

We thank you for your continued consideration of our manuscript and appreciate the constructive feedback from Reviewer 3. We are grateful for the reviewer’s thoughtful comments, which have helped us to improve the clarity and scientific rigor of our manuscript. Below we provide a point-by-point response to each of the suggestions.

Reviewer #3:

Comment: **Clarify and tighten the opening sentence**

**Original: Respiratory disorders are among the most common causes of emergency presentations in small animal practice.**

**Suggested revision: Respiratory disorders represent a frequent cause of emergency presentation in small animal veterinary practice.**

Response: We appreciate the reviewer’s suggestion to improve clarity in the opening sentence. As advised, we have revised the sentence to be more concise and direct.

Revised text (Lines 51–52): “Respiratory disorders represent a frequent cause of emergency presentation in small animal veterinary practice.”

Comment: **Strengthen the contrast between human and veterinary medicine**

**Original: In contrast, veterinary medicine currently lacks disease-specific scoring systems for respiratory disorders.**

**Suggested revision: Despite the widespread use of prognostic scoring systems in human respiratory medicine, veterinary medicine lacks analogous, disease-specific tools for assessing the prognosis of respiratory disorders.**

Response: Thank you for highlighting the need to better emphasize the contrast between human and veterinary medicine. We have incorporated the suggested revision to strengthen this comparison.

Revised text (Lines 68–70): “Despite the widespread use of prognostic scoring systems in human respiratory medicine, veterinary medicine lacks analogous, disease-specific tools for assessing the prognosis of respiratory disorders.”

Comment: **Clarify and streamline main findings (Opening paragraph)**

**Original: In this study, we demonstrated that several parameters of physical examination and blood test were related with prognosis.**

**Suggested revision: This study demonstrated that several physical examination findings and blood test parameters were associated with prognosis in dogs with respiratory disorders.**

Response: We thank the reviewer for pointing out the importance of clearly presenting the main findings. We have adopted the suggested wording to enhance clarity and readability.

Revised text (Lines 253–254): “This study demonstrated that several physical examination findings and blood test parameters were associated with prognosis in dogs with respiratory disorders.”

Comment: **Eliminate redundancy and combine similar ideas (Phosphate findings)**

**You repeat the association between elevated phosphate and prognosis multiple times. Here’s a cleaner version of that section:**

**Revised summary paragraph (merging lines 253–259 and 263–265):**

**Multivariate analysis identified elevated plasma phosphate concentration as an independent prognostic indicator in canine respiratory disorders. This association remained significant even in subgroup analyses, suggesting phosphate may serve as a generalizable biomarker across different respiratory etiologies. While hyperphosphatemia is a known prognostic factor in chronic kidney disease and other critical conditions in both human and veterinary medicine, our findings highlight its potential role in acute respiratory disease as well.**

Response: We agree with the reviewer that the section on phosphate findings was redundant. Accordingly, we have condensed and integrated the relevant content to avoid repetition and to improve logical flow.

Revised text (Lines 254–260): “Multivariate analysis identified elevated plasma phosphate concentration as an independent prognostic indicator in canine respiratory disorders. This association remained significant even in subgroup analyses, suggesting phosphate may serve as a generalizable biomarker across different respiratory etiologies. While hyperphosphatemia is a known prognostic factor in chronic kidney disease and other critical conditions in both human and veterinary medicine, our findings highlight its potential role in acute respiratory disorder as well.”

Comment: **Polish scientific phrasing and condense speculative language**

**Original: Although many causes of increasing blood phosphate level were considered in this study, circulatory insufficiency and transcellular shift might be the main causes of blood phosphate elevation.**

**Suggested revision: Among various possible causes of elevated phosphate, circulatory insufficiency and transcellular shifts are likely the primary contributors in our cohort.**

Response: Thank you for this insightful recommendation. We have modified the sentence to present a more scientifically appropriate and concise explanation, following your suggestion.

Revised text (Lines 268–270): “Among various possible causes of elevated phosphate, circulatory insufficiency and transcellular shifts are likely the primary contributors in our cohort.”

Comment: **Improve clarity in linking clinical signs to mechanisms**

**Original: Since tendency of decrease in body temperature, heart rates and increase in blood lactate concentration were observed in Non-Survivors, it was considered that circulatory insufficiency and severe hypoxia were occurred in Non-Survivors.**

**Suggested revision: The observed decreases in body temperature and heart rate, along with elevated lactate levels in non-survivors, support the presence of circulatory insufficiency and severe hypoxia in these cases.**

Response: We appreciate the reviewer’s effort to improve the clarity of our interpretation of the clinical signs. We have revised the sentence to better articulate the link between observed signs and the proposed pathophysiological mechanisms.

Revised text (Lines 271–274): The observed decreases in body temperature and heart rate, along with elevated lactate levels in non-survivors, support the presence of circulatory insufficiency and severe hypoxia in these cases.

Comment: **Remove unnecessary repetition about human studies and phosphate**

**You repeat references to COPD, CURB-65, and phosphate in humans multiple times. Streamline with this:**

**Revised version:**

**In human medicine, elevated phosphate levels have been linked to poor outcomes in diseases such as COPD and pneumonia, including those assessed using scoring systems like CURB-65 [15,16]. Similar associations have been reported in critical illness and burn patients, where hyperphosphatemia often results from acute kidney injury and tissue breakdown [17,18].**

Response: We acknowledge the repetition in referencing human studies related to phosphate. We have revised the section to present these findings more concisely, as suggested.

Revised text (Lines 264–268): “In human medicine, elevated phosphate levels have been linked to poor outcomes in diseases such as chronic obstructive pulmonary diseases and pneumonia, including those assessed using scoring systems like CURB-65 [15,16]. Similar associations have been reported in critical illness and burn patients, where hyperphosphatemia often results from acute kidney injury and tissue breakdown [17,18].”

Comment: **Shorten and refine limitations section**

**Your limitations are well laid out but overly wordy. Here's a tighter version of the first point:**

**Original: Firstly, only cases with radiographically confirmed respiratory abnormalities were included. As a result, certain conditions such as functional disorders or early-stage diseases without apparent radiographic changes may have been excluded.**

**Suggested revision: First, the study included only cases with radiographic evidence of respiratory disease, potentially excluding early-stage or functional disorders.**

Response: We appreciate the reviewer’s feedback on improving the clarity and conciseness of the limitations section. We have rephrased the initial point in line with your recommendation.

Revised text (Lines 350–352): “First, the study included only cases with radiographic evidence of respiratory disease, potentially excluding early-stage or functional disorders.”

We have carefully addressed all comments and incorporated the suggested revisions into the manuscript. All changes have been clearly highlighted in the revised version. We hope that these revisions meet your expectations and that our manuscript is now suitable for publication in PLOS ONE. Thank you once again for your time and the constructive feedback.

Sincerely,

Toshiyuki Tanaka, DVM, PhD

Osaka Metropolitan University

Laboratory of Veterinary Advanced Diagnosis and Treatment

1-58, Rinku-oraikita, Osaka city, Osaka, 598-8531, Japan

email: f21724w@omu.ac.jp
